# Covid-19: did higher trust societies fare better?

**DOI:** 10.1007/s44155-023-00035-3

**Published:** 2023-03-20

**Authors:** Bernard H Casey

**Affiliations:** 1 SOCial ECONomic RESearch, London, UK; 2 SOCial ECONomic RESearch, Frankfurt, Germany

## Abstract

Social trust**—**between governments and people and between individuals—and trust in science were proposed as prerequisites for tackling covid. Others suggested less democratic societies were more able to impose strict rules stopping the virus. These propositions were tested for a group of mainly advanced countries.

The dependent variable is cumulated deaths from covid. Findings are broken down between (a) OECD member countries, (b) these and countries having cooperation agreements with it, and (c) all these plus China. They are also broken down by time—between (a) the period before the appearance of “new variants” in late 2020 and (b) the period from then until end September 2021.

The best, most parsimonious, models explain nearly half of the changes in the level of deaths. Trust in government improves outcomes, as does interpersonal trust. Vaccine antipathy does not play a role. Also, there is little indication that authoritarian regimes performed better than higher trust societies. In the first period, increasing wealth inequality—indicating a more divided society—is related to higher death rates. Hospital bed availability is important then, but not thereafter. Furthermore, as the pandemic persisted, the importance of pre-existing levels of social trust declined.

The paper warns that institutions and cultures cannot easily be transferred from one country to another. Nor would all transfers be desired. It also suggests that some other lessons of what contributed to better outcomes under covid might be relevant for the monkeypox virus—its successor public health emergency.

## Introduction

The question of whether some societies were better at coping with the covid pandemic was asked at an early stage of the crisis. One commentator suggested that authoritarian societies might have an advantage: *If developed democracies cannot pull together to stop the spread of the virus, manufacture the goods the world needs to fight it, and make plans to jumpstart the global economy, China will do so. On the other end of this crisis, the result could be a decisive global shift toward its authoritarian model* [29]. In the same vein, another asked: *Has populism made coronavirus worse?*, and pointing to how *the US and the UK lead the death tolls, while Russia and Brazil are catching up quickly*, noted that *many observers have drawn a line between populism and coronavirus fatality. If you put Donald Trump, Boris Johnson, Jair Bolsonaro and Vladimir Putin in a pod you get a populist plague* [33]. Closely related to this was the question posed by analysists concerned with societal trust. They wanted to know: *Do low-trust societies do better in a pandemic?*, and they reminded their readers: *When you don’t trust your neighbours to wash their hands, you stand farther away*. Examples of some of the (south) eastern European states were picked out [16]. The topic did not go away. At the end of September 2020, trust—between individuals, between them and health care systems, and between all of these parties and governments was emphasised as being crucial to successful containment of the virus [1, 25, 41]. In November, the UK’s Behavioural Insights Team (the “nudge unit”) emphasised social trust, and it urged the relevant authorities to *measure it and make it an integral part of economic policy making* [6].

Two years and more into the pandemic it becomes worthwhile asking whether higher trust societies did, indeed, fare better. After prolonged periods in which societies have been subjected to varying degrees of control on social interaction, which has had its own consequences for economic performance, policy makers have changed tack. Controls have been removed. In the most extreme case—for example, the UK—people were told that restrictions were lifted for once and for all. This was “freedom day”. Nevertheless, they were urged to exercise “personal responsibility” to make sure that they behaved in ways that did not encourage transmission—including respecting social distancing and mask wearing. Personal responsibility could be understood as a component of social capital and trust, not a contradiction to it. This was made clear by one commentator who, asking whether social networks increase or decrease the covid contagion rate, quoted the case of Sweden. There, the prime minister had said, “The only way to manage this crisis is to face it as a society, with everyone taking responsibility for themselves, for each other and for our country” [9]. Or, as a philosopher put it in a more general discussion of the concept, “An appreciation for the role of social capital in community life may lift some outcomes out of the moral paradigm of individual and personal responsibility and place them in the paradigm of collaborative or collective responsibility” [26].

The remainder of this paper is divided as follows. First, literature on the contribution of social capital to combating covid is reviewed. Second, an examination of the link between social capital and covid-induced deaths is made using statistical analysis. This covers two periods—the earlier phase of covid and the period from late 2020 into autumn 2021, when the newest variants of covid were becoming apparent. Third, some conclusions are drawn about whether social capital might prove useful in diminishing the impact of future public health emergencies.

## What we know so far

Although the term “social capital” [43] was not used by all of them, many commentators have made reference to it. In short, social capital involves a mixture of trust, norms and social networks that both “improve the efficiency of society by facilitating coordinated outcomes” [42] and describe the “connections amongst individuals—social networks and norms of reciprocity and trustworthiness that *arise from them*” [43], my emphasis.^1^ An attempt to provide a glossary of the components of social capital that contribute to different health outcomes was constructed as early as 2003 [3], but this document did not make any specific recommendations. Also drawing from the early 2000s interest in Putnam’s work, it was suggested that the importance it placed upon networks and trust meant that enhancing social capital could, of itself, contribute to improving public health [55]. A large-scale review of the contribution of social capital to health outcomes was published in 2020. It found that of the 454 separate associations that a total of 155 studies had looked at, 217 found higher social capital to be positively associated with better health, 16 found a negative association with it, and the remainder showed either no association at all (141) or were inconclusive (24) [50].

As early as 2005, the World Health Organisation issued a set of guidelines on dealing with disease outbreaks. This referred to the importance of “public trust and compliance” and to their role in securing “appropriate public participation to support [the] rapid containment” of an outbreak [58]. These views were reiterated in amore general paper on “The Role of Public Trust During Pandemics” a decade later [51]. In the context of the AIDS pandemic, a number of studies considered the contribution of social capital to mitigating the spread of that condition [45]. Social capital was also assessed as a potential for controlling the A(H1N1) influenza pandemic in 2009 (Rönnerstrand [49]).

Nevertheless, the concept of social capital was criticised for its conceptual vagueness [2]. This, in turn, has resulted in uncertainty as to which specific components of social capital should be promoted and when they might be effective. An examination of “Trust, Social Capital, Civil Society and Democracy” made the point that there is no one to one correspondence, either at an individual or at a societal level between the two components of social capital—political trust and social trust. It also suggested that what both represent is perceptions about society as much as about the individual [37]. A meta review of evidence stressed the importance of “what, who, where, when, why and how” and concluded that it was “difficult to disentangle every component of SC [social capital] and assess whether a certain dimension, setting, or level for SC is linked with better health outcomes than others” [17].

The outbreak of the covid pandemic gave opportunity for the production of a number of academic studies that attempted to throw light on whether there was any substance to the arguments of the importance of social capital in stemming both the spread of the disease and its consequences in terms of lives lost. These studies looked at interactions between individuals, but they also acknowledged the role of a sense of community and an acceptance of the strictures of those in authority—be they clinicians, medical experts, or governments as coordinators. Some of them were summarised in two reviews of the literature, which organised them under the headings of implementation of policies, compliance with rules, perceptions of risk and trust in science, and consequences, including whether the pandemic itself led to behavioural changes, and mortality [13, 60]. It is mortality that is the subject of this paper.

The two reviews reported only one study that was concerned with mortality. It showed a negative relationship between the number of deaths and the trust in institutions across some 25 European Union (EU) countries [39].^2^ However, both reviews covered only papers published during the early months of the pandemic. In the interim, the number of subsequent analyses of the contribution of trust, social capital and political systems to covid-related developments has increased. Some of these analyses have been limited to individual countries, and others have been concerned with behavioural patterns at the individual level.^3^ Nonetheless, cross-country, macro analyses remain few and far between.

A comparison across counties (subdivisions of states) in the USA, admittedly in the early months of the pandemic, suggested that the spread of the disease was faster the higher the level of social capital. On the other hand, with respect to the level of fatalities, the reverse was the case [7]. A second cross-county analysis in the USA found a positive relationship between social capital and vaccination take-up. Social capital seemed more important in rural areas than in urbanized areas. There were also indications that some forms of social capital—as measured by membership of religious associations—might have had a negative impact on take-up [44]. A third American cross-county comparison looked at the total number of cases, the weekly growth in cases, and the number of deaths [34]. It found a negative relationship between the level of social capital and each of these three outcomes.

A comparison between prefectures in Japan found that infection rates were lower in those where social capital was higher. However, social capital alone was insufficient to mitigate high infection rates in prefectures where population density was high and opportunities for social distancing were more limited [30]. A comparison of districts within the Hubei province of China recorded a low correlation between, respectively, social trust and collective efficacy and exposure to covid, but a stronger, and negative correlation was found between political trust and, especially, strong social networks and exposure to the disease [52, 60].^4^

A cross-country study concerned not with deaths but with transmission, and so with the number of infections, found peak transmissions occurred more quickly in countries where some indicators of trust were high [35]. This apparently paradoxical result was a consequence of social capital having negative as well as positive dimensions—after all, social capital is high in mafia-like organisations [22, 57]. Where people do trust one another, they mix together more freely, and so infection is passed on faster. In fact, a U-shaped relationship was found. Both low levels of trust and high levels of trust were associated with early peaking in transmission rates. High trust in political institutions was also associated with an early peaking of infections. If people trust what they are being told, they are more likely to observe the new rules of behaviour that are being imposed to slow down transmission.

A second cross-country study, which claimed to cover a high proportion (some 86 per cent) of the world’s population, looked at the speed at which deaths from covid doubled [18]. It covered the period until the end of July 2020, and it used a number of variables that represented social capital—specifically, trust in government, trust amongst people, affiliation to membership groups, and civic engagement. On top of this, it included income distribution as an explanatory variable. Both trust in government and trust of others appeared to produce the expected outcomes—the higher their levels, the lower the speed of spreading. However, affiliation to groups and civic engagement accelerated the incidence of deaths—a finding that is not surprising given that both imply mixing. Societies that were less equal on the basis of income disparities were also societies in which deaths rose faster.

A third cross-country study considered the relationship between cumulative deaths by the start of 2021 and the level of both interpersonal trust and trust in government. The higher the former, the lower the level of deaths. With respect to the latter, no real differences could be found [5].

A fourth study, including 177 countries, and one that attracted widespread attention, looked at determinants of covid-related deaths.^5^ It found that “measures of trust in the government and interpersonal trust, as well as less government corruption, had larger, statistically significant associations with lower standardised infection rates. High levels of government and interpersonal trust, as well as less government corruption, were also associated with higher COVID-19 vaccine coverage among middle-income and high-income countries where vaccine availability was more widespread, and lower corruption was associated with greater reductions in mobility” [11].

A fifth study, including 107 countries, found three factors of importance in mitigating covid deaths—respectively, trust in government, trust in society and trust in science [47]. Here the authors found that trust in society actually had a negative impact—something they ascribed to the way in which, where high trust between people did exist, confidence about the behaviour and health status of those with whom they were mixing lead people to consider the use of personal protective measures as less important.^6^ Of the two positive factors, trust in science was by far the most important.

Last, in another paper, the number of deaths was used as an explanatory variable in a cross-country study of the extent to which government policy with respect to covid was trusted. The higher deaths were, the less governments were trusted [48].

There are suggestions that social capital might sometimes have a malignant effect—it can lead to over-confidence and people interacting with those they should not. However, most of the studies reviewed indicate that higher levels of social capital and/or of trust between people or between governments and their populations does have a beneficial effect. It limits the number of deaths or the number of transmissions.

## Examining the relationship between trust and mortality

This paper looks at whether higher trust/social capital produces better covid outcomes, whereby “better” means a lower number of deaths (deaths per million in the population) experienced. It concentrates on deaths directly associated with covid, rather than “excess deaths”, even though some have argued the latter to be a better measure [10]. It does so because covid deaths are widely and frequently reported, whereas excess deaths are less readily available and require more explanation. In short, deaths are immediately visible, and their reporting is something that is more likely to provoke a behavioural response. Infections/confirmed cases have also been widely reported. However, these are increasingly unreliable as a consistent measure [53].^7^

Given that trust in government has declined in some countries since the onset of the pandemic, it is important to look at measures of trust/social capital that were collected before the outbreak of covid—in other words, measures that could be treated as purely exogenous and have not been influenced by experience of the pandemic itself.^8^ In the absence of a single indicator of social capital, a variety of proxies, drawn from a number of published sources, were used.^9^ These were:

- From the World Values Survey (WVS) 2017–2020 wave—trust in government, trust in people with whom we normally have contact, coupled with more synthetic indicators such as particular trust, general trust, and the “radius of trust”^10^;
- From the OECD—trust in government (reported for 2017);
- From the Hofstede Model of Cultural Dimensions data set—the extent to which people are individualistic;
- From the Standardized World Income Inequality Database—the extent of income disparities as measured by the Gini coefficient (from the late 2000 and teens);
- From the World Bank—the extent of wealth disparities as measured by the Gini coefficient (ditto);
- From the World Health Organisation and the Wellcome Trust—the extent to which people trust vaccinations (from 2015 and 2018), which is also often seen as a proxy for trust in political and medical elites [23],^11^
- From the World Bank data base—the number of hospital beds (per 1,000 people), which is an indicator of the health service infrastructure in each country; and
- From the Economist Intelligence Unit—a measure of how “democratic” governments are (from 2019).

Each of the variables represents a level—for example, the proportion of the population trusting government. The scale runs from 0 to 100.

The cumulated number of deaths was taken from the Johns Hopkins University database on covid as reported in Our World in Data. This records deaths per million in the population.

The initial dataset comprised some 44 countries. These were selected, largely, because they had a level of trust in government reported by the OECD. That listing includes some countries that are not OECD members, but it does not include China. The WVS data set did include the latter country. However, the number of countries for which data from the latest WVS wave are available is more limited. Earlier data is likely to be less representative of views prevailing in the late 2010s [10].^12^ The study used WVS values but generated its own up-to-date values by using principal components analysis in conjunction with measures provided by the Gallup World Poll. The correlation between the variables for the countries used in the Covid-19 E study and those in the WVS data set was 0.91, and for those in the OECD data set it was 0.85 (this coefficient is bounded by − 1 and 1, whereby 0 means no relationship at all).

From the WVS data set, a total of 35 countries could be drawn. These included four non-OECD countries—Brazil, China, Indonesia, and Russia. For the 34 countries for which data from both sources were drawn, the correlation between the OECD and the WVS measures of trust in government was high but by no means perfect—it was only about + 0.79 and for those that were OECD members, it was about + 0.72.

In the remaining investigations the WVS rather than the OECD indicator of trust in government was used. In general, its use produced more robust, if not substantially different results from when the OECD indicator was used. The countries selected are relatively homogenous—and so they were different from those in some of the larger studies that included developing economies as well as more advanced economies [11, 17, 47, 57]. The OECD is commonly regarded as “a club of mostly rich countries”. Brazil works with the OECD and is categorised as an emerging market economy, as is China. But so, too, are several OECD member countries, including Colombia, Hungary, Mexico, Poland, and Turkey.^13^

To take account of the overall level of economic wellbeing in the countries in the sample, an additional variable—gross domestic product per head at purchasing power parity—was used in the modelling. It might be assumed that better-off countries had more financial resources at their disposal to help them deal with the pandemic. In addition, given that contracting covid is more likely to prove fatal for older rather than younger people, the proportion of the population aged 65 and over was also included as an explanatory variable. A further background statistic, that of the level of obesity within a country, was added to the data set on the grounds that it has been recognised as a factor that increases the likelihood of death amongst those who have been infected by covid.

Last, because the deaths to be explained are a cumulative total, the point in time when a notable number of deaths were first reported—in excess of one per 100 thousand (10 per million)—was recorded.

A table providing descriptive statistics is given in the annex—Appendix Table 6.

The modelling was conducted using ordinary least squares regression.

### Results for the first period (up to mid-november 2020)

Initial analysis was carried out for the period up until mid-November 2020. This was the period before any of the “new” variants of covid had been identified and before vaccines had become publicly available.

Bivariate analysis was employed to check initial correlations. The results are reported in Table 1.

**Table 1:** Bivariate analysis—regressions on deaths for first period

	Coefficient	S.e	t-stat	R squared	Sample
Not involving trust					
gdp per head	− 63.6299	112.4485	− 0.57	0.0096	35
Pop aged 65 or more	− 6.06723	10.22412	− 0.59	0.0106	35
Obesity	70.6443	35.42178	1.99	0.1076	35
Hospital beds per 1000	− 42.6197	16.96403	− 2.51	0.1606	35
Elements of trust					
Non democratic	− 232.766	176.1467	− 1.32	0.0503	35
Wealth gini	10.36109	5.640172	1.84	0.0928	35
Income gini	23.92623	10.15779	2.36	0.1439	35
Trust indicators					
Trust in government	− 7.51868	2.431766	− 3.09	0.2246	35
Most people trusted	− 4.49023	2.35592	− 1.91	0.0992	35
Trust radius	− 2.26284	4.711531	− 0.48	0.0072	34
Trust radius squared	− 0.0125	0.036567	− 0.34	0.0036	34
Combined radius				0.0418	34
Anti vax	3.810266	5.483863	0.69	0.0153	33
Individualistic	0.743764	2.575871	0.29	0.0031	29

The t-statistic should be not too far below two for significance; R-squared gives the variance explained by the model (as a %); sample is number of countries for which data is available. Data refer to the first period—up to mid-November, 2020

The variables with high explanatory powers were inserted into the multiple regression. Those that proved to be statistically insignificant were then dropped.

The regression showed that there is a positive impact of trust in government on the number of deaths experienced (the negative coefficient indicates that the higher the number of deaths, the lower the level of trust and vice versa). This finding holds regardless of whether the sample is restricted to OECD countries or to all countries except China.

In order to check whether “authoritarian” regimes did better, a variable indicating regimes that were “less than democratic” was introduced.^14^ This contributed nothing to the outcome in any of the models in which it was tested.^15^

Tests using radius of trust, which attempted to see if too much trust produced negative consequences, were tried. Although the variables produced indications that this could be the case, the models using them produced fits no better than those merely using the extent to which most people were trusted.^16^

The extent to which people were “individualistic” had no impact upon outcomes.^17^

Income inequality was important. However, what was more important was wealth inequality. Societies with a high level of wealth inequality are often considered to be divided/low trust societies. If they are, this measure of social capital/trust had the expected impact. Moreover, the coefficient it yielded was highly significant.

Scepticism about vaccines appeared, at first sight, to be associated with a higher level of deaths, but the trust in vaccinations variable contributed nothing to the model.

The number of hospital beds available was important. In addition, better-off countries did perform better. However, the variable GDP per head was not statistically significant and it was dropped from the analysis.

The variable giving the proportion of the population aged 65-plus also yielded no significant contribution to the model. Levels of obesity (adjusted, adult body-mass index) by country also added nothing.

Last, the length of time before the first deaths were reported contributed slightly to the analysis, although primarily to the models that included China.^18^

The best models showed that around 47 to 49 per cent of the changes in the number of deaths could be explained by changes in the level of trust in government, the feeling that most people could be trusted, and the extent of inequality in wealth distribution. This is shown by the R^2^ statistic in Table 2.

**Table 2:** Regression results for the first period

	All in sample				OECD members				All excluding China			
	Coeff.	S.e	t stat	Prob t	Coeff.	S.e	t stat	Prob t	Coeff.	S.e	t st	Prob t
Trust in govt	− **7.66**	**2.32**	− **3.31**	**0.00**	− **6.41**	**3.52**	− **1.82**	**0.08**	− **9.24**	**2.72**	− **3.39**	**0.00**
Most people trusted	− 2.41	2.02	− 1.19	0.24	− 4.48	2.52	− 1.78	0.09	− 2.53	2.01	− 1.26	0.22
Wealth gini	**11.56**	**5.03**	**2.30**	**0.03**	**14.82**	**5.99**	**2.48**	**0.02**	**13.11**	**5.21**	**2.51**	**0.02**
Beds	− **26.60**	**15.29**	**− 1.74**	**0.09**	− 26.14	17.03	− 1.53	0.14	− 24.07	15.42	− 1.56	0.13
Constant	− 14.83	391.97	− 0.04	0.97	− 217.39	443.75	− 0.49	0.63	− 83.54	395.68	− 0.21	0.83
R sqd	0.486				0.472				0.486			
R bar sqd	0.417				0.391				0.416			
Prob F	**0.000**				**0.002**				**0.001**			
Obs	35				31				34			

The probability of t shows the probability that the value of the coefficient does not, in fact, equal zero. Thus, 0.03 is a probability of 3%.Normally, variables are accepted when then probability of their being equal to zero is under 5%, and sometimes under 10%. Coefficients where significance is 95% or more have the t-statistic in bold. The F statistic, and so the probability of F, shows whether the linear regression model provides a better fit to the data than a model that contains no independent variables. The probability of 0.002, thus means that the probability that the variables do not show anything extra is something is 0.2%

Excluding China and/or excluding non-OECD countries changed the results little.

The regressions are summarised in Table 2. The most parsimonious models are presented—in other words, the regressions containing insignificant variables that scarcely contributed to model fit are excluded.^19^

Models were checked to ensure they involved no multicollinearity—using the Variance Inflation Factor (VIF) statistic. Equally, tests for heteroscedasticity—using the Breusch-Pagan test—showed this not to be present.

### Results for the second period (mid-november 2020 to end september 2021)

Data on cumulated deaths for the period from mid-November 2020 to the end of September 2011 was used.^20^ This end date comes not long after the WHO had recognised the Delta variant of covid as the dominant strain, but it is well before the onset of Omicron.^21^ The same regressors were employed as with the model for the earlier period. The results are shown in Table 3.

**Table 3:** Regression results for the second period

	All in sample				OECD members				All excluding China			
	Coeff.	S.e	t stat	Prob t	Coeff.	S.e	t stat	Prob t	Coeff.	S.e	t stat	Prob t
Trust in govt	− **15.06**	**5.90**	− **2.55**	**0.02**	− 12.00	9.32	− 1.29	0.21	− **17.09**	**7.04**	− **2.43**	**0.02**
Most people trusted	− **12.63**	**5.13**	− **2.46**	**0.02**	− **15.28**	**6.67**	− **2.29**	**0.03**	− **12.79**	**5.20**	− **2.46**	**0.02**
Wealth gini	− 8.74	12.82	− 0.68	0.50	− 7.18	15.84	− 0.45	0.65	− 6.75	13.48	− 0.50	0.62
Beds	5.01	38.94	0.13	0.90	0.43	45.08	0.01	0.99	8.25	39.86	0.21	0.84
Constant	**2565.31**	**997.99**	**2.57**	**0.02**	2462.56	1174.29	2.10	0.05	**2477.41**	**1022.90**	**2.42**	**0.02**
R sqd	0.434				0.384				0.411			
R bar sqd	0.359				0.289				0.329			
Prob F	**0.002**				**0.011**				**0.003**			
Obs	35				31				34			

Notes as for Table 2

The bold items are those that are significant at 95%

What is to be noted is that the explanatory power (the R^2^) of the model is little changed. The statistical significance of the trust in government variable becomes less important—at least in OECD countries. Wealth inequality ceases to be important. However, trust between people becomes more important and is statistically significant (at 95 percent) in each sub-group of countries for the first time. The number of hospital beds completely ceases to be a significant explanatory variable.

### Results for the whole period

Last, the model was applied to the whole period up until end September 2021. The results are shown in Table 4. Both the trust in government variable and the trust in other people variable are statistically significant. As in for the second period, the wealth inequality variable is not significant. Nor are the number of hospital beds. The explanatory power of the model is higher for the whole than for the first period and as high as it was for the second period.

**Table 4:** Regression results for the entire period

	All in sample				OECD members				All excluding China			
	Coeff.	S.e	t stat	Prob t	Coeff.	S.e	t stat	Prob t	Coeff.	S.e	t stat	Prob t
trust in govt	− **22.72**	**7.01**	− **3.24**	**0.00**	− **18.40**	**10.88**	− **1.69**	**0.10**	− **26.33**	**8.32**	− **3.16**	**0.00**
most people trusted	− **15.04**	**6.10**	− **2.46**	**0.02**	− **19.76**	**7.79**	− **2.54**	**0.02**	− **15.32**	**6.14**	− **2.49**	**0.02**
wealth gini	2.82	15.24	0.18	0.86	7.64	18.49	0.41	0.68	6.36	15.92	0.40	0.69
beds	− 21.58	46.29	− 0.47	0.64	− 25.70	52.62	− 0.49	0.63	− 15.83	47.08	− 0.34	0.74
constant	**2550.48**	**1186.33**	**2.15**	**0.04**	2245.17	1370.87	1.64	0.11	**2393.87**	**1208.24**	**1.98**	**0.06**
R sqd	0.478				0.426				0.457			
R bar sqd	0.408				0.337				0.382			
prob F	**0.001**				**0.005**				**0.001**			
obs	35				31				34			

Notes as for Table 2

The bold items are those that are significant at 95%

### Simplifying the model yet further

In the regressions reported in Tables 3 and 4, a number of the explanatory variables lose their statistical significance—notably the measure of wealth equality and the number of hospital beds. Therefore, the equations were tested again—this time leaving out these two variables. For the second period and the whole period, the explanatory power of the model was reduced, but not by a large amount. In other words, in the second period, and over the whole period, social trust mattered, but only in the first period was physical health infrastructure (number of hospital beds) important. Results are given in Appendix 2. In Table 5, the R^2^ values are presented.

**Table 5:** R^2^ values for alternative model specifications

	All countries	OECD countries	All countries except China
Models used so far			
First period	0.486	0.472	0.486
Second period	0.502	0.458	0.479
Both periods	0.540	0.495	0.521
Models using only trust in govt and trust in others			
First period	0.246	0.167	0.222
Second period	0.422	0.378	0.401
Both periods	0.471	0.409	0.448

### The predictive power of the model

Whilst the models appear to provide an explanation in general, they are less good at indicating what happened country-by-country. Interestingly, none of the earlier papers reported on above provided comparisons of predictions with the actual outcomes—even though they were using county-by-county or country-by-country data and, so, this would have been possible. Their objective had been to provide aggregate results rather than to explain what had happened in a particular administrative entity.

Appendix 3 indicates that for some countries, actual and predicted values were relatively close. For example, for Chile, France, Poland, Portugal, Slovenia and Russia, projections were always within plus or minus 10 per cent of the actual values. On the other hand, in some cases, the predictions were wildly out. Deaths in countries such as Australia, Iceland, Japan, Korea, and New Zealand were heavily over-predicted. The over-prediction for China is small in terms of absolute numbers but large in relative terms.

Except for China, each of the countries for which large over-prediction occurred is an island (Korea’s only land border is with the north). China became, effectively, a closed country with the onset of the pandemic. However, in all these countries, “closure” was a response and, thus, should be treated as an endogenous variable, not as an exogenous determinant. When a variable indicating “closed” was included in the model, it had no explanatory power in the models covering the initial period. It did so only for the second period—underlining the role of “closure” as a response. Appendix 4 shows this.

## Conclusions

At least in terms of deaths from covid, some societies appear to have performed better than others. Their ability to do so might have been assisted by the higher level of trust and/or social capital that they possess. This paper started with the presumption that there existed no relationship between trust/social capital and performance, and that the examples cited by commentators might better be explained by ethnographic rather than statistical analysis. It finished by suggesting that some relationship might, after all, be discernible. In this respect, it provided some support both to propositions made with respect to the speed at which the infection spread and to those made with respect to deaths that were consequent upon infection.

Nevertheless, the results should be treated with caution. First, they are sensitive to the indicator of trust used. Using some measures did allow the construction of a model showing an impact but using other measures did not. Second, many of the indicators of trust/social capital that have been suggested from other analyses failed to show their importance when included in the analysis carried out here. That they have shown importance in other analyses might be because these referred to a wider range of countries than were included in this study.

Third, trust is not fixed for all times [24]. During covid it changed, and it changed in relation to outcomes and to the perceived performance of governments and agencies in their handling of the pandemic. In some countries, there is evidence that it increased during the early months of the pandemic. Examples included Finland [32], Austria [28], Italy, the Netherlands, Canada, Germany, and New Zealand [4]. In others, there is evidence that it fell. Examples here are the UK [4, 14, 15, 20, 27], France [28], (Becuwe [4]), Israel [36], Spain, Japan, Sweden, Brazil, Poland, and the USA [4]. Longer follow-throughs, covering more than the earlier months, are not available. At best, it can be reported that, by 2022, trust in government in general had reached its lowest level ever [40]. There are suggestions that, in societies where political cleavage was deep-rooted, deterioration in trust was more intensive—for example, in the cases of France, Brazil, Poland, the UK, and the USA [4]. It was also suggested that, when governments were fresh and new, as was the case in Austria, trust in them increased [28]. Thus, even if trust and social capital are important, the extent to which societies can learn from the findings of this paper are also limited. What generates high levels of trust in societies is, in part, the product of history and culture. However, levels of trust can also be lost rapidly and, equally, they can be built up only slowly.

Whilst testing for relationship, it was clear that “outlying” cases might have a distorting impact on the outcomes. Tests for the impact of outliers were made. China might be presumed to be a special case. It has one of the highest levels of trust in government, one of the highest levels of trust in other members of society, one of the lowest levels of individualism, and one of the highest levels of trust in vaccinations. China also reported the lowest level of deaths from covid. It also ranks top amongst those countries labelled less than democratic.^22^ However, even excluding China, which is an outlier on almost all dimensions, the alternative hypothesis—that trust/social capital did have a beneficial impact—could be maintained.

Although the models selected were able to show outcomes “in general”, they were not good at showing them “in particular”. It is not possible to use the models to explain outcomes in specific countries, merely to use them to suggest that trust and social capital are, potentially, of importance.

If other countries had trust/social capital levels such as appear to exist in China, global outcomes to the outbreak of the pandemic might have been different. Yet few countries are like China, and it is unlikely that they could be made like it—or that their inhabitants would wish their countries to be so. On the other hand, the paper also shows that switching from democratic to authoritarian forms of government would not have improved outcomes. That, at least, provides some comfort—even if it contradicts some of the assertions promulgated by earlier commentators.

Last, it might be tempting to suggest that findings of this investigation into performance during covid could be extended to assess performance in other cases when societies are faced by an infectious disease, the spread of which is changing fast. For example, when in 2022 the WHO decreed monkey pox to be “a public health emergency of international concern”, its secretary-general pointed out that its transmission “can be stopped with the right strategies in the right groups” [56]. Or, as one commentator said, “[a] robust, acceptable, and sustainable monkeypox response would need to recognise and address the structural drivers of disease emergence, including social, cultural, and ecological factors” [46].

Because monkeypox is an infection overwhelmingly reported as occurring in men who have sex with men, and especially in those with multiple sexual partners, it might be tempting to try to draw lessons from the earlier AIDS crisis and efforts to understand whether social capital prevented HIV transmission. However, many of the AIDS studies focused on sub-Saharan Africa. Far fewer looked at advanced industrialised countries [46].

More importantly, whatever might have been learnt from the experience of the covid crisis, one thing stands out. That is that the foundations on which any counter-offensive built upon trust in government, and/or on trust in other people, might be substantially weaker now than they were at the onset of the pandemic. Indeed, the experiences during the covid crisis might well have contributed to their weakening.

## Data Availability

All data used for this analysis are drawn from publicly available websites. The dataset generated during analysis is available from the corresponding author on reasonable request.

## Appendices

### Appendix 1

See Table 6.

**Table 6:** Descriptive statistics for variables used in analysis

Variable	Mean	sd	Min	Max
Deaths in first period	334.1121	294.9105	3.209	872.154
Deaths in second period	916.7523	715.8916	0.001	2791.629
Deaths in both periods	1250.864	885.6638	3.21	3113.089
WVS trust in government	38.61143	18.58959	11.9	94.6
Score on how democratic	0.085714	0.284029	0	1
WVS most people trusted	35.39143	20.68217	4.5	73.9
Radius of trust	59.28693	11.19118	39.76971	92.36291
Radius of trust squared	3636.499	1444.489	1581.63	8530.906
Prop. individualistic	55.24138	22.94972	13	91
Antivax rate	13.55758	9.464969	3	45.2
Wealth gini	73.52286	8.669591	49.8	90.2
Income gini	47.49429	4.676156	33.4	53.7
Natural log of GDP per capita	10.33639	0.454342	9.38023	11.34545
Beds	4.514286	2.772676	1	13
Obesity rate	26.18857	1.36915	22.7	28.9
Prop. aged 65 or over	17.22857	4.994619	6	28

Sample: all countries used in analysis. Not all countries had values for all variables

### Appendix 2

See Tables 7, 8, 9.

**Table 7:** Regression results for period 1

	All in sample				OECD members				All excluding China			
	Coeff.	s.e	t stat.	Prob. t	Coeff.	s.e	t stat.	Prob. t	Coeff.	s.e	t stat.	Prob. t
Trust in govt	− 6.57	2.63	− 2.50	0.02	− 6.29	4.06	− 1.55	0.13	− 7.23	3.04	− 2.38	0.02
Most people trusted	− 2.27	2.36	− 0.96	0.34	− 2.20	2.97	− 0.74	0.47	− 2.32	2.39	− 0.97	0.34
Constant	668.03	113.53	5.88	0.00	647.86	138.03	4.69	0.00	691.32	126.12	5.48	0.00
R sqd	0.246				0.167				0.222			
R bar sqd	0.199				0.107				0.171			
Prob F	0.011				0.078				0.021			
Obs	35				31				34			

Notes as for Table 2

**Table 8:** Regression results for period 2

	All in sample				OECD members				All excluding China			
	Coeff.	s.e	t stat.	Prob. t	Coeff.	s.e	t stat.	prob. t	Coeff.	s.e	t stat.	prob. t
Trust in govt	− 15.97	5.59	− 2.86	0.01	− 12.59	8.59	− 1.47	0.15	− 18.16	6.43	− 2.82	0.01
Most people trusted	− 12.71	5.02	− 2.53	0.02	− 16.05	6.28	− 2.55	0.02	− 12.89	5.07	− 2.54	0.02
Constant	1983.32	241.45	8.21	0.00	1993.11	292.21	6.82	0.00	2060.75	266.96	7.72	0.00
R sqd	0.422				0.378				0.401			
R bar sqd	0.385				0.333				0.362			
prob F	0.000				0.001				0.000			
obs	35				31				34			

Notes as for Table 2

**Table 9:** Regression results for periods 1 and 2

	All in sample				OECD members				All excluding China			
	Coeff.	s.e	t stat.	Prob. t	Coeff.	s.e	t stat.	Prob. t	coeff.	s.e	t stat.	prob. t
Trust in govt	− 22.54	6.61	− 3.41	0.00	− 18.87	10.12	− 1.87	0.07	− 25.39	7.60	− 3.34	0.00
Most people trusted	− 14.98	5.94	− 2.52	0.02	− 18.25	7.40	− 2.47	0.02	− 15.21	5.99	− 2.54	0.02
constant	2651.35	285.71	9.28	0.00	2640.98	344.25	7.67	0.00	2752.07	315.36	8.73	0.00
R sqd	0.471				0.409				0.448			
R bar sqd	0.438				0.367				0.412			
Prob F	0.000				0.001				0.000			
Obs	35				31				34			

Notes as for Table 2

### Appendix 3

See Table 10.

**Table 10:** Actual and predicted values for full period (values refer to deaths per million)

Country	Actual	Full model		SC variables only	
		Predicted	% Diff	Predicted	% Diff
*All countries*					
SVK	2319	1556	− 33	1811	− 22
DEU	1099	1242	13	1262	15
POL	2001	1821	− 9	1918	− 4
CHE	1272	306	− 76	364	− 71
CZE	2840	2047	− 28	2096	− 26
ISL	89	1025	1046	1013	1032
JPN	140	1019	628	1367	876
GBR	2005	1548	− 23	1473	− 27
HUN	3134	1404	− 55	1543	− 51
TUR	753	1196	59	1117	48
KOR	49	836	1618	1134	2230
NOR	158	272	73	205	30
NZL	5	685	12900	705	13282
ITA	2169	1845	− 15	1855	− 14
AUT	1217	1012	− 17	1089	− 11
SWE	1463	753	− 49	570	− 61
FRA	1731	1641	− 5	1710	− 1
AUS	51	1239	2338	1291	2439
EST	1024	1334	30	1357	32
USA	2101	1616	− 23	1451	− 31
NLD	1062	925	− 13	736	− 31
PRT	1768	1804	2	1811	2
DNK	457	758	66	608	33
MEX	2130	2467	16	2305	8
ESP	1849	1633	− 12	1616	− 13
CHL	1950	1981	2	1847	− 5
SVN	2194	2046	− 7	2091	− 5
GRC	1430	2400	68	2444	71
FIN	202	686	240	660	228
BRA	2790	2468	− 12	2271	− 19
CHN	3	− 480	− 15039	− 400	− 12569
COL	2464	2682	9	2541	3
IDN	514	1243	142	1081	110
LTU	1856	1302	− 30	1425	− 23
RUS	1395	1373	− 2	1316	− 6
*OECD countries*					
SVK	2319	1536	− 34	1811	− 22
DEU	1099	1216	11	1262	15
POL	2001	1793	− 10	1918	− 4
CHE	1272	330	− 74	364	− 71
CZE	2840	2021	− 29	2096	− 26
ISL	89	910	916	1013	1032
JPN	140	945	575	1367	876
GBR	2005	1531	− 24	1473	− 27
HUN	3134	1412	− 55	1543	− 51
TUR	753	1446	92	1117	48
KOR	49	824	1594	1134	2230
NOR	158	242	54	205	30
NZL	5	664	12,497	705	13282
ITA	2169	1840	− 15	1855	− 14
AUT	1217	945	− 22	1089	− 11
SWE	1463	756	− 48	570	− 61
FRA	1731	1644	− 5	1710	− 1
AUS	51	1160	2183	1291	2439
EST	1024	1359	33	1357	32
USA	2101	1646	− 22	1451	− 31
NLD	1062	929	− 12	736	− 31
PRT	1768	1881	6	1811	2
DNK	457	660	44	608	33
MEX	2130	2547	20	2305	8
ESP	1849	1569	− 15	1616	− 13
CHL	1950	2119	9	1847	− 5
SVN	2194	1996	− 9	2091	− 5
GRC	1430	2414	69	2444	71
FIN	202	590	193	660	228
COL	2464	2757	12	2541	3
LTU	1856	1302	− 30	1425	− 23
*All countries excluding China*					
SVK	2319	1488	− 36	1811	− 22
DEU	1099	1275	16	1262	15
POL	2001	1853	− 7	1918	− 4
CHE	1272	164	− 87	364	− 71
CZE	2840	2121	− 25	2096	− 26
ISL	89	1000	1017	1013	1032
JPN	140	1005	617	1367	876
GBR	2005	1553	− 23	1473	− 27
HUN	3134	1377	− 56	1543	− 51
TUR	753	1077	43	1117	48
KOR	49	791	1524	1134	2230
NOR	158	178	13	205	30
NZL	5	577	10860	705	13282
ITA	2169	1849	− 15	1855	− 14
AUT	1217	1006	− 17	1089	− 11
SWE	1463	711	− 51	570	− 61
FRA	1731	1647	− 5	1710	− 1
AUS	51	1212	2283	1291	2439
EST	1024	1301	27	1357	32
USA	2101	1649	− 22	1451	− 31
NLD	1062	920	− 13	736	− 31
PRT	1768	1782	1	1811	2
DNK	457	750	64	608	33
MEX	2130	2530	19	2305	8
ESP	1849	1649	− 11	1616	− 13
CHL	1950	1985	2	1847	− 5
SVN	2194	2094	− 5	2091	− 5
GRC	1430	2453	72	2444	71
FIN	202	640	218	660	228
BRA	2790	2546	− 9	2271	− 19
COL	2464	2769	12	2541	3
IDN	514	1091	112	1081	110
LTU	1856	1264	− 32	1425	− 23
RUS	1395	1375	− 1	1316	− 6

### Appendix 4

See Tables 11, 12.

**Table 11:** Regression results for the first period including “closed” as a variable

	All in sample				OECD members				All excluding China			
	Coeff	s.e	t stat	Prob t	Coeff	s.e	t stat	Prob t	Coeff	s.e	t stat	Prob t
Trust in govt	− 7.03	2.41	− 2.92	0.01	− 6.24	3.50	− 1.78	0.09	− 8.85	2.71	− 3.27	0.00
Most people trusted	− 1.83	2.10	− 0.87	0.39	− 3.53	2.64	− 1.34	0.19	− 1.79	2.07	− 0.86	0.40
Wealth gini	9.61	5.43	1.77	0.09	12.48	6.29	1.98	0.06	10.93	5.43	2.01	0.05
Beds	− 23.71	15.59	− 1.52	0.14	− 21.34	17.44	− 1.22	0.23	− 19.45	15.66	− 1.24	0.22
Closed	− 120.43	124.00	− 0.97	0.34	− 149.78	130.77	− 1.15	0.26	− 162.37	125.78	− 1.29	0.21
Constant	91.68	407.38	0.23	0.82	− 86.49	455.67	− 0.19	0.85	40.21	402.78	0.10	0.92
R sqd	0.502				0.499				0.515			
R bar sqd	0.416				0.398				0.429			
Prob F	0.001				0.003				0.001			
Obs	35				31				34			

Notes as for Table 2

**Table 12:** Regression results for the second period inc closed

	All in sample				OECD members				All excluding China			
	Coeff	s.e	t stat	Prob t	coeff	s.e	t stat	prob t	Coeff	s.e	t stat	Prob t
Trust in govt	− 9.24	4.75	− 1.94	0.06	− 10.57	6.69	− 1.58	0.13	− 14.15	5.19	− 2.73	0.01
Most people trusted	− 7.25	4.15	− 1.75	0.09	− 7.30	5.04	− 1.45	0.16	− 7.14	3.97	− 1.80	0.08
Wealth gini	− 26.86	10.71	− 2.51	0.02	− 26.91	12.02	− 2.24	0.03	− 23.30	10.39	− 2.24	0.03
Beds	31.76	30.77	1.03	0.31	40.80	33.33	1.22	0.23	43.22	29.98	1.44	0.16
Closed	− 1117.14	244.72	− 4.56	0.00	− 1261.08	249.85	− 5.05	0.00	− 1229.99	240.80	− 5.11	0.00
Constant	3553.29	803.97	4.42	0.00	3564.68	870.62	4.09	0.00	3414.78	771.13	4.43	0.00
R sqd	0.671				0.695				0.695			
R bar sqd	0.614				0.634				0.640			
Prob F	0.000				0.000				0.000			
Obs	35				31				34			

Notes as for Table 2

## Footnotes

1 The relationship between trust and social capital might be less automatic than some think and might even be erroneous. Moreover, the relationship between these two elements might, itself be dependent upon the country and culture in which the observation is made. See, here, [52].

2 Institutional trust was measured by respondents’ trust in five institutions, namely, parliament, politicians, political parties, the police, and the legal system.

3 On how the pandemic indicated a very high level of institutional trust expressed by people in Italy with respect to the public authorities’ ability to deal with the COVID-19 emergency, and how this contrasted with relatively low levels of institutional trust in that country, [19]. A somewhat similar picture emerged in Korea, where trust in society, people, and the central and local governments improved substantially, even if trust in the judicature, the press, and religious organizations fell, [31]. A study comparing counties (subdivisions of states) within the United States found that reductions in mobility in order to comply with social distancing advice and regulations were positively related to social capital, [7].

4 “Social trust” referred to people’s willingness to help their neighbours and the belief that neighbours can be trusted. “Collective efficacy” referred to the willingness of individuals to intervene in cases of anti-social behaviour, whilst “social networks” referred to individuals’ knowledge of what other people in their neighbourhood were doing.

5 It was even reported in *Private Eye*, No 1567 of 18 February to 3 March, 2022.

6 Similar results were suggested with respect to a qualitative study caried out in Singapore in the early stages of the pandemic [59].

7 Reported deaths are immediately visible. Longer-term mortality following, for example, long covid and “complications” associated with it feed into excess deaths but not reported deaths.

8 The level of trust in some governments did, indeed, fall because of its handling of covid [4]. This matter is taken up in more detail later.

9 The American study [34] has a single indicator, but this is actually a composite made up from a variety of sub-indicators relating to “family structure and stability, family interaction and investment, civil society, trust and confidence in institutions, community cohesion, institutions, volunteerism, and social organisation”. The Japanese study [30] uses a “social capital comprehensive index” recorded in the Cabinet Office.

10 This is a synthetic measure constructed by [35], using WVS data. It defines the circle of people amongst whom trust is operative.

11 This is a more specific indicator than the “trust in science” variable used in the study by [47].

12 The study by [17] did include data from earlier waves of the WVS. It also used a variable to indicate when these were employed. It showed that data collected from these earlier WVS waves did have an impact on the outcomes found.

13 This listing uses the International Monetary Fund’s categorisation.

14 Those countries scoring as “flawed democracies”, or below that level, were singled out.

15 A study looking at whether authoritarian regimes were better able to impose mobility restrictions than more democratic ones found this not to be the case [21].

16 This outcome is to be contrasted with the studies emphasising the” double edged nature of trust” [47, 59].

17 In one study, looking at Switzerland, this characteristic was found to be important in determining the extent to which people perceived risks to their own health of the pandemic and, thus, to the extent to which they were willing to accept measures imposed by the authorities to control the spread of covid [51].

18 This unimportance of the start date for when deaths were first registered is explained largely by the concentration of the period in which that start date did occur. China reported its first deaths at end January 2020. The next country reported deaths nearly four weeks later, whilst the last of the other 31 countries reported deaths nearly nine weeks later. The average (and median) distance after China was rather over six weeks. Data was not reported consistently. Some of the first reports were of higher levels of death than the one per hundred thousand used as the start level.

19 The parsimonious nature of the models avoided any problems that the data was over-fitted—too many independent variables relative to the number of observations [38].

20 This was the same cut-off period used in the paper by [10].

21 See https://www.who.int/emergencies/diseases/novel-coronavirus-2019/media-resources/science-in-5/episode-45---delta-variant.

22 [60] makes specific reference to the special case of China. It notes “In democratic societies, social capital may be especially important as compliance cannot be ensured by more forceful means. In authoritarian China, however, collective actions may depend less on social capital, but more on forceful means Accordingly, different forms of social capital may play differential roles in shaping the COVID- 19 response, and they may also exert differential effects in authoritarian China than in Western democracies.”.
